# Evaluating the efficiency, productivity change, and technology gaps of China’s provincial higher education systems: A comprehensive analytical framework

**DOI:** 10.1371/journal.pone.0294902

**Published:** 2024-01-19

**Authors:** Jiani Liu, Kim Jungyin, Shim Jaewoo, Lee Heechul, Wasi Ul Hassan Shah

**Affiliations:** 1 Department of English Education, Jeonbuk National University, Jeonju, Republic of Korea; 2 School of Management, Zhejiang Shuren University, Hangzhou, China; 3 Department of Economics, University of Religions and Denominations, Qom, Iran; National Kaohsiung University of Science and Technology / Industrial University of Ho Chi Minh, TAIWAN

## Abstract

China’s higher education system is one of the largest and most complex in the world, with a vast number of higher education institutions scattered across different provinces. Evaluating the efficiency, productivity change, and technology gaps of these institutions is significant for understanding their performance and identifying areas for improvement. In this context, this study employs three different approaches, DEA super-SBM, Malmquist Productivity Index, and Meta-Frontier Analysis, to evaluate the efficiency, productivity change, and technology gaps of China’s provincial higher education systems. The study results revealed that the average higher education efficiency in China is 1.0015 for the study period of 2010–2021. A rapid and continuous increase was witnessed in higher education efficiency in China from 2014 to 2020. Meta-frontier and Group-frontier, higher education efficiency scores of low-level literate provinces are greater than middle and high-level literate provinces. However, the TGR of higher and middle-level literate provinces is greater than low-level literate provinces, indicating a superior technological level. The average MI score is 1.0034, indicating growth in productivity change. Efficiency change is the main determinant in higher education productivity growth instead of technological growth. The Middle and Low-level literate provinces witnessed growth in higher education productivity, while high-level literate provinces observed a decline in productivity change. The Kruskal-Wallis test provides evidence that a significant statistical difference exists among the three groups of education levels for the average scores of MI, EC, TC, and TGR.

## 1. Introduction

The 2030 Agenda for Sustainable Development, adopted by all UN members in 2015, promotes peace and prosperity for humanity and Earth. The 17 Sustainable Development Goals (SDGs) are at the heart of this agenda, calling for action from all countries, regardless of development level, in a global collaboration. These goals recognize that eradicating poverty and other types of deprivation requires comprehensive initiatives to improve healthcare, education, inequality, and economic growth. They also emphasize climate change and marine and forest protection [1, 2].

Developing countries have demonstrated exceptional dedication to promoting and improving educational infrastructure in line with United Nations goals. Building schools in remote regions has been a priority so that all kids, especially those from disadvantaged backgrounds, have the chance to get a basic education. Governments have instituted programs to improve the quality of education by investing in teacher development, revising curricula, and introducing new approaches to education that promote active participation and realistic application [3–5]. Girls’ education has been singled out as a focus of measures designed to level the gender gap in the workplace. Many developing countries invest in adult education and vocational training to better prepare their citizens for the workforce and boost their economic growth. Initiatives like mobile learning and online education platforms are accepted to reach remote and neglected groups despite infrastructure limitations. These combined efforts show how determined developing countries are to meet UN education goals, which will pave the path for long-term prosperity, eliminating poverty, and more equitable communities [6, 7].

Moreover, technology and economic growth of any economy depend on the quality of higher education in that country. Higher education promotes research, innovation, and specialized training, creating a talented workforce that contributes to research innovations. Scientists, engineers, and academics investigate new technologies at universities and colleges. Their innovations open the doors to new sectors, products, and services, boosting economic growth and competitiveness [8–10]. Higher education also helps to impart technology usage. Engineering and computer science graduates help progress technology across sectors. Higher education facilitates industry-academia collaboration, sharing knowledge and technology. University-business partnerships promote research, entrepreneurship, and innovation commercialization, boosting social prosperity. A strong higher education system attracts investment and fosters technological innovation by ensuring a skilled workforce pool. It fosters a knowledge-based economy that relies on research, innovation, and development to be competitive [11–13]. Higher education needs efficient resource utilization. Institutions can educate more students by maximizing infrastructure, faculty, technology, and finance. In developing countries with limited resources, effective resource use helps institutions to accommodate more students while maintaining educational standards, closing the access gap. Optimizing resources ensures a favorable learning environment, including well-maintained infrastructure and enhanced facilities students require to complete their education [14, 15]. Faculty allocation and workload optimization improve instructional capacity, student-teacher ratios, academic support, and customized attention. Resource efficiency maximizes funds and reduces costs. These funds research, scholarships, and faculty development, improving higher education. Higher education resource efficiency improves access, quality, learning environments, faculty capacity, and funding allocation. Institutions advance education and society through optimal resource usage [16–18].

China has one of the world’s largest and most diverse higher education systems, with more than 2,900 universities and colleges. The Chinese government has invested significantly in higher education in recent years to make Chinese universities more competitive globally [19]. Chinese higher education needs efficient resource allocation. By properly distributing resources, infrastructure, faculty, and research support, China has improved education quality and accessibility in recent years. Strategic allocation allows the building and refurbishment of campuses, classrooms, and libraries to meet a growing student population. Appropriate funding ensures research, scholarships, faculty recruitment, and creative teaching methods, developing academic excellence and recruiting talented people [20]. Effective faculty allocation and support ensure qualified teachers through appropriate ratios and professional development, benefiting the education system. Research and innovation boost academic prestige, economic prosperity, and scientific and technological progress. Allocating resources to impoverished regions helps bridge the educational gap between urban and rural areas. China’s higher education development benefits from resource allocation that funds infrastructure, research, faculty development, and regional differences. By enhancing higher education quality and access, resource allocation improves China’s socioeconomic development and global competitiveness [21, 22]. Higher education efficiency has been evaluated in China for some particular year or region [23–25], but a comprehensive higher education performance evaluation is lacking.

Our study aims to address this research gap and evaluate the efficiency, productivity change, and technology gaps in China’s provincial higher education systems using a comprehensive approach. We use the DEA Super-SBM model to measure educational resource efficiency over 12 years from 2010 to 2021 for 31 provinces, analyze the technology gap ratio through Meta-frontier analysis, and estimate educational productivity change with the Malmquist productivity Index. Finally, we employ the Kruskal-Wallis test to assess the statistically significant differences among different Chinese regions based on their education levels. The rest of the study is structured as follows: Section 2 presents a comprehensive literature review, Section 3 outlines the methodologies employed, and Sections 4 and 5 present the results, discussions, and conclusion, respectively.

## 2. Literature review

DEA has been extensively used to gauge the efficiency and total factor productivity growth in different sectors and industries around the globe [26–29]. Recent developments in DEA methodology allowed researchers to use more advanced and efficient models to gauge the efficiency and productivity change in different kinds of DMUs [30–32]. However, the application of DEA in the education sector is insufficient and scarce [33]. The following three sections explain the recent literature on efficiency and productivity change in educational sectors of different countries around the globe.

Due to the growing higher education system and the need for more resources to accommodate enrolment, efficiency evaluation in higher education has received attention. Universities’ diversified goals, diffuse decision-making, and complex production processes make productivity and efficiency more difficult to define and adopt [34]. In scenarios involving centralized decision-making for multiple units, resource allocation becomes a critical challenge. Studies introduce a novel approach that utilizes Data Envelopment Analysis (DEA) for the allocation of fixed resources among these units, such as banks, supermarkets, and public universities. Xiong et al. [35] proposed a parallel DEA-based method that treats individual periods (e.g., years) as parallel divisions, effectively addressing resource allocation competition. The resulting allocation of multi-period resources is found to be acceptable to both central management and the evaluated units. As a practical application, the study evaluated the R&D performance of 61 public universities in China and reallocated government funding resources among them. Chen et al. [36] assessed resource utilization efficiency in universities, a crucial aspect of achieving balanced development strategies. Universities often share resources, including faculty and assets for teaching and research. While previous assessments relied on radial measures within Data Envelopment Analysis (DEA), this research introduced non-radial measures and considered both internal and external evaluations. Using aggregated two-stage DEA models, the study evaluated the efficiency of 52 Chinese universities in 2014 from both perspectives. Key findings include relatively high average operating efficiency according to both internal and external evaluations, with approximately 53% of universities deemed efficient.

Ding et al. [37] introduced an innovative Data Envelopment Analysis (DEA) approach that considers shared, fixed-sum inputs within a network DEA model. They divided scientific research activities into two interrelated subsystems, both dependent on shared government grant funding. Factoring in the impact of this shared input on performance, the study calculated the minimum input adjustments needed to establish a common equilibrium efficient frontier. Total efficiency was then determined by aggregating subsystem efficiencies. The application of their models to assess Chinese universities reveals diverse overall efficiency outcomes, offering valuable insights and recommendations for central policymakers. Further, a model extends the DEA meta-frontier framework by allowing the assessment of mixed-type DMUs without requiring identical DMUs. The study used the example of assessing the scientific research efficiency of faculty members at Inner Mongolia University to illustrate our model. This model intended to offer a fair and equitable method for performance evaluation, one that accurately reflects the actual performance of mixed-type DMUs [38].

Wang et al. [39] assessed scientific research efficiency in 10 Chinese urban regions with a focus on Chengdu-Chongqing. It used Data Envelopment Analysis (DEA) to measure and compare university research input and output efficiency. Findings reveal a slight improvement in research efficiency from 2016 to 2020, with variations between agglomerations. Research-oriented universities within Chengdu-Chongqing face misalignment of research themes, funding, and human resources. Authors argue that efforts to enhance efficiency are needed, with scale having minimal impact. Excessive research investment is a primary cause of inefficiency. Mirasol-Cavero and Ocampo [40] enhance university department efficiency assessment, particularly in the presence of imprecise, missing, or vague data. It introduces Fuzzy Preference Programming—DEA (FPP-DEA) as a more flexible approach compared to existing methods that rely on fuzzy set theory in Data Envelopment Analysis (DEA). In FPP-DEA, inputs are expressed as precise values, while outputs are represented as fuzzy numbers. A case study in a prominent Philippine university illustrates the effectiveness of this approach. The findings demonstrate that this model can calculate efficiency even when data is missing or uncertain, with data completeness leading to more precise efficiency scores. Ding et al. 2021 [41] focused on evaluating the performance of individual departments within a university or research institution. Unlike previous research, it takes into account the heterogeneity among these departments. To achieve this, the study employs data envelopment analysis (DEA) models designed for non-homogeneous decision-making units (DMUs) within a two-stage network structure. The first stage pertains to faculty research, while the second concerns student research. The authors divide each department into distinct input and output subgroups based on their homogeneity within both stages. An additive DEA model is introduced to assess the overall efficiency of these non-homogeneous DMUs within the two-stage network structure. Empirical findings offer insights to help universities enhance the research performance of individual departments and the institution as a whole. Except for China, numerous research studies gauge the efficiency, productivity change, and heterogeneity in educational sectors in other parts of the globe [38, 42–64].

## 3. Methodology

The assessment of efficiency among decision-making units in a specific industry typically involves the application of two well-known methodologies: parametric Stochastic Frontier Analysis (SFA) and nonparametric Data Envelopment Analysis (DEA). In SFA, the production frontier is estimated by fitting a stochastic production function that regresses observed outputs against inputs. The coefficients within this production function signify the efficiency with which inputs are utilized to generate outputs, helping to identify sources of inefficiency in the production process. Conversely, DEA evaluates the relative efficiency scores of individual decision-making units using multiple input-output pairs. In our study, we employ the DEA Super-SBM Model to assess the efficiency of higher education in 31 Chinese provinces. We also utilize the Meta frontier approach to analyze the technology gap ratios across different groups, while the Malmquist Productivity Index (MI) is applied to measure changes in higher education productivity across these provinces. We further investigate the primary determinants of productivity changes, specifically focusing on technology and efficiency changes. To test for statistically significant differences among three educational level groups, we employ the Kruskal-Wallis test, examining average MI, EC, TC, and TGR scores.

### 3.1 DEA super SBM model

Tone [65] introduced the super-efficiency SBM model, designed for assessing the efficiency of uniform decision-making units. This non-radial DEA model allows for the concurrent examination of inputs and outputs, thus enhancing the precision of efficiency assessment. Unlike the radial DEA model, the super-efficiency SBM model includes slack variables in the estimation process, thereby circumventing its constraints. This particular model can effectively differentiate among efficient DMUs.

Assume that there are η DMUs, each with its own input and output set. Three vectors can represent these inputs and outputs: $x\in R^{M},y^{g}\in R^{S1},y^{b}\in R^{S1}$. These scalars represent the output that can be anticipated from S_1_ for a given m unit input. It’s worth emphasizing that X>0, Y^g^>0, Y^b^>0 are all positive numbers that exceed zero. The set of potential outcomes for production in this scenario is as follows: The input matrix, X, can be written: $X=[x_{1},x_{2},\ldots ,x_{N}]\in R^{N\times M}$, while the resulting output matrix, Y^g^, can be written as $Y^{g}=[y^{g1},y^{g2},\ldots ,y^{gN}]\in R^{S1\times N}$.


$$P=\{(x,y^{g},y^{b})|,x\geq X\eta |,y^{g}\leq Y\eta |,y^{b}\geq Y\eta |,\eta \geq 0\}$$ (1)

The predicted production in Formula (1) is less than the ideal predicted output at the border. If there is any wiggle room in the DMU evaluation (represented by (x_0_y^g^_0_,y^b^_0_), Tone’s SBM model accounts for it by considering the production possibility set.


$$\gamma =min(\frac{1-\frac{1}{M}\sum_{i=1}^{M}\frac{S_{i}^{-}}{x_{io}}}{1+\frac{1}{S1+S2}(\sum_{r=1}^{S1}\frac{S_{r}^{g}}{y_{r0}^{g}}+\sum_{r=1}^{S2}\frac{S_{r}^{b}}{y_{r0}^{b}})})$$ (2)

$$s.t.\left\{ \begin{array}{c} x_{0}=X\eta +S^{-}\\ y_{0}^{g}=Y^{g}\eta -S^{g}\\ y_{0}^{b}=Y^{b}\eta +S^{b}\\ S^{-}\geq 0,S^{g}\geq 0,S^{b}\geq 0,\eta \geq 0 \end{array}\right.$$

Formula (2) stands for the DMU’s Efficiency, and the variational value can be any integer between 0 and 1. The symbols represent input, output, and slack (S^−^S^−^,S^g^,S^b^). Only when the technical efficiency is *γ* is 1 and S^−^,S^g^,S^b^ are all 0, is the DMU at the forefront of production; otherwise, the DMU is inefficient. The Charnes-Cooper transformation can be used to convert the nonlinear Eq (2) into a linear model.


$$\kappa =m(T-\frac{1}{M}\sum_{i=1}^{M}\frac{S_{i}^{-}}{x_{io}})$$ (3)

$$\left\{ \begin{array}{c} 1=T+\frac{1}{S1+S2}(\sum_{r=1}^{S1}\frac{S_{r}^{g}}{y_{ro}^{g}}+\sum_{r=1}^{S2}\frac{S_{r}^{b}}{y_{ro}^{b}})\\ x0T=X\beta +S^{-}\\ y_{0}^{g}T=Y^{g}\beta -S^{g}\\ y_{0}^{b}T=Y^{b}\beta +S^{b}\\ S^{-}\geq 0,S^{g}\geq 0,S^{b}\geq 0,\beta \geq 0,T\geq 0 \end{array}\right.$$

There are situations, though, in which specific decision-making bodies are also effective at evaluating the technological viability of alternatives. The super-efficiency SBM model (Super SBM model) was established by extending prior work to create a fair method for efficiency measurement.

$$\gamma^{*}=m\left[\frac{\frac{1}{M}\sum_{i=1}^{M}\frac{\bar{x}i}{\frac{x}{0}0}}{\frac{1}{S1+S2}(\sum_{r=1}^{S1}\frac{{\bar{y}}_{r}^{s}}{y_{ro}^{g}}+\sum_{r=1}^{S2}\frac{{\vec{y}}_{r}^{b}}{y_{ro}^{b}})}\right]$$ (4)

$$\left\{ \begin{array}{c} \bar{x}\geq \sum_{j=1,\neq 0}^{N}\eta_{j}x_{j}\\ {\bar{y}}^{g}\leq \sum_{j=1,\neq 0}^{N}\eta_{j}y^{g}{}_{j}\\ {\vec{y}}^{b}\geq \sum_{j=1,\neq 0}^{N}\eta_{j}y^{b}{}_{j}\\ \bar{x}\geq x0,{\bar{y}}^{g}\leq y_{0}^{g},\vec{y^{b}}\geq y_{0}^{b},{\bar{y}}^{g}\geq 0,\eta \geq 0, \end{array}\right.$$

Super-efficiency of DMU is denoted by γ* In Formula (4), it can be more than 1.

### 3.2 Meta frontier analysis

The Meta-frontier Model enhances the assessment of group DMU efficiency. It necessitates comparing DMUs within the same group to confirm the presence of similar technology. The Technological Gap Ratio (TGR) plays a key role in evaluating the technological progress within a group. TGR quantifies the technology disparities between one group and the meta frontier [66, 67].


$$TGR=\frac{MHEE}{GHEEi}$$ (5)

GHEE*i* quantifies the efficiency of higher education institutions within a distinct group category, whereas MHEE assesses their Meta-Higher Education Efficiency (Meta-HEE) at a designated technology level. As proposed by Chiu et al. [68], the TGR (Technology Gap Ratio) employs a distance-based metric to ascertain how close a meta-frontier technology is to a group’s frontier technology. A TGR value of 1 signifies the absence of a technological gap between the group and the Meta frontier, rendering it a prevalent regional differentiating statistic.

### 3.3 Malmquist Productivity Index

In the analysis of Total Factor Productivity (TFP) changes, the Malmquist index is decomposed through the utilization of the Data Envelopment Analysis (DEA) method. This approach offers substantial support for subsequent studies within the field. By employing a distance function to depict a production process involving multiple inputs and outputs, this method bypasses issues related to subjective weight assumptions, as it does not necessitate the use of any specific type of production function [69–71].

The Malmquist productivity index proves highly valuable for examining productivity and its influencing factors, particularly when dealing with panel data that consists of multiple observations across different time periods in the evaluation of Decision-Making Units (DMUs). Widely applicable and straightforward to implement, the DEA technique is capable of calculating and decomposing the Malmquist index, a metric used to assess changes in Total Factor Productivity (TFP) [72]. The DEA method for efficiency estimation hinges on determining the production frontier based on the available data and then positioning the Decision-Making Unit (DMU) within this production envelope [73–75].

For the evaluation of Higher Education productivity under input orientation and Variable Returns to Scale (VRS), Cappellesso et al. [76] treat each Chinese province as an individual DMU. They suggest that this approach is suitable for a specific set of K provinces *K*(*k* = 1,2…). In their paper, Fare et al. [77] define the output distance function on the output set *S*_*t*_, as the mapping from inputs *x*∈*R*+^*N*^ to outputs *y*∈*R*+^*M*^ for each time step period *T* = 1,…,*T*. To specify this function, we define:

$$S^{t}=\{[x^{t},y^{t}]:x^{t}\;\mathrm{can}\;\mathrm{produce}\;y^{t}\}$$ (6)

Any function of the production set *S*^*t*^, such as the vector *y*^*t*^, will result in a value for this distance function that is less than or equal to 1. A non-member of the production set *S*^*t*^ Will have a distance function value larger than one.

*M*_0_(*x*^*t*+1^,*y*^*t*+1^,*x*,*y*) is the geometric mean of the two Malmquist indices, indicating the Malmquist index between t and t+1 [77].


$$M_{0}(x^{t+1},y^{t+1},x,y)=\sqrt{\frac{D_{0}^{t}(x^{t+1},y^{t+1})}{D_{0}^{t}(x^{t},y^{t})}\frac{D_{0}^{t+1}(x^{t+1},y^{t+1})}{D_{0}^{t+1}(x^{t},y^{t})}}$$ (7)

Based on the level of technology in period *t*, the production efficiency is denoted by the functions $D_{0}^{t}(x^{t},y^{t})$ and $D_{0}^{t}(x^{t+1},y^{t+1})$. Similarly, the production technology efficiency levels of periods *t* and *t*+1.are represented by $D_{0}^{t+1}(x^{t},y^{t})$ and $D_{0}^{t+1}(x^{t+1},y^{t+1})$, respectively. The Malmquist index has been deconstructed by Fare et al. [77] into its constituent parts, technical efficiency change, and technical change."

$$\begin{array}{l} M_{0}(x^{t+1},y^{t+1},x,y)=\sqrt{\frac{D_{0}^{t}(x^{t+1},y^{t+1})}{D_{0}^{t}(x^{t},y^{t})}\frac{D_{0}^{t+1}(x^{t+1},y^{t+1})}{D_{0}^{t+1}(x^{t},y^{t})}}\\ =\frac{D_{0}^{t+1}(x^{t+1},y^{t+1})}{D_{0}^{t}(x^{t},y^{t})}\sqrt{\frac{D_{0}^{t}(x^{t},y^{t})}{D_{0}^{t+1}(x^{t},y^{t})}\frac{D_{0}^{t}(x^{t+1},y^{t+1})}{D_{0}^{t+1}(x^{t+1},y^{t+1})}}\\ \frac{D_{0}^{t+1}(x^{t+1},y^{t+1})}{D_{0}^{t}(x^{t},y^{t})}\;(4) \end{array}$$ (8)

Illustrates the connection between TEC and the production frontier. The technology-enabled gap (TEC) is determined by the percentage variance between the current production level and the maximum attainable through technology. The realization of this potential at the current technical level is contingent upon the effective coordination of various resource components.


$$\sqrt{\frac{D_{0}^{t}(x^{t},y^{t})}{D_{0}^{t+1}(x^{t},y^{t})}\frac{D_{0}^{t}(x^{t+1},y^{t+1})}{D_{0}^{t+1}(x^{t+1},y^{t+1})}}$$ (9)

Presents an explanation of technical change (TC) and its impact on the technological frontier in relation to productivity. Technical change refers to advancements in technology that lead to a shift in the production frontier, creating the potential for higher output using the same inputs. This implies that technological progress contributes to enhanced production efficiency.

### 3.4 Kruskal–Wallis test

In cases where there are more than two separate groups, the Kruskal-Wallis test becomes a valuable tool for assessing statistical significance [78]. This test is instrumental in identifying notable statistical distinctions among the three groups of Chinese provinces concerning average MI, EC, TC, and TGR. The following hypotheses are put forth:

H_01_: The distribution of MI is the same across categories of different groups.

H_02_: The distribution of EC is the same across categories of different groups.

H_03_: The distribution of TC is the same across categories of different groups.

H4: The distribution of TGR is the same across categories of different groups.

### 3.5 Data sources and variables selection

Input-output selection in the DEA evaluation has its distinct significance as it could impact the estimated efficiency and productivity change scores [79, 80]. Numerous studies employed the DEA to evaluate higher education efficiency in different countries. Based on existing literature, Table 1 illustrate the inputs and outputs selection for efficiency and productivity change estimation. Data from 31 Chinese provinces and administrative units were collected for the years 2010–2021 from the National Bureau of Statistics of China, China Statistical Yearbook, Provincial Statistical Yearbook, China Statistical Yearbook on Science and Technology, Educational Statistics Yearbook of China, China Statistical Yearbook on High Technology Industry and Ministry of Education of the People’s Republic of China.

**Table 1:** Inputs and outputs.

Inputs/Outputs	Measurement
1. Schools	Number of Schools
2. Faculty members	Person
3. R&D Personnel	Person
4. Funding received by higher education institutions	100 million yuan
5. Government expenditures for higher education	100 million yuan
6. Expenditure on R&D by higher education	100 million yuan)
7. Fixed Assets Investment on Higher Education infrastructure	100 million yuan
8. Registered Students in Higher Education Schools	Person
9. Average Students receiving higher education	Per 100000 population
10. Science Papers publications	(piece)
11. Publication on S&T	(kind)

## 4. Results and discussion

Table 2 and S1 Fig detail the illiteracy rates in 31 provinces of China, along with their respective ranges and regions. We divided all provinces and administrative units into three groups based on literacy rate. The first column contains the names of the provinces. The " Illiteracy " column displays the percentage of each province’s illiterate population. The range of illiteracy rates is indicated in the "Range" column. It specifies minimum and maximum illiteracy rates for each province. The "Regions" column classifies the provinces according to their literacy rates. It categorizes the general literacy level in each province into three categories: High level-literate, Middle level-literate, and Low level-literate. Provinces classified as "High-level literate" have low illiteracy rates, from 0.5% to 3.0%. It indicates that a substantial portion of the population in these provinces is highly literate. The illiteracy rates of provinces in the "Middle-Level literate" category range from 3.1% to 5.0%. It indicates that the population has a moderate level of literacy. The provinces classified as "Low-Level literate" have the highest prevalence of illiteracy, ranging from 5.1% to 35%. It suggests that these provinces have a comparatively low level of literacy. This table provides an overview of the illiteracy rates in various provinces and their classification based on their literacy levels, allowing for a comparative analysis of literacy across country regions. We found that Beijing is highly literate, with the lowest illiteracy rate of 0.79, and Tibet is considered the least literate, with an illiteracy rate of 34.27%.

**Table 2:** Regional division of China based on literacy.

Province	Illiteracy	Range	Groups
Beijing	0.79%	0.5–3.0	High-level literate
Liaoning	1.11%	0.5–3.0	
Jilin	1.31%	0.5–3.0	
Chongqing	1.46%	0.5–3.0	
Tianjin	1.66%	0.5–3.0	
Shanxi	1.68%	0.5–3.0	
Guangdong	1.87%	0.5–3.0	
Shanghai	1.90%	0.5–3.0	
Hebei	2.02%	0.5–3.0	
Heilongjiang	2.15%	0.5–3.0	
Hunan	2.24%	0.5–3.0	
Hubei	2.38%	0.5–3.0	
Fujian	2.50%	0.5–3.0	
Jiangxi	2.55%	0.5–3.0	
Henan	2.81%	0.5–3.0	
Guangxi	2.82%	0.5–3.0	
Jiangsu	3.04%	3.1–5.0	Middle-Level literate
Shaanxi	3.38%	3.1–5.0	
Xinjiang	3.47%	3.1–5.0	
Zhejiang	3.55%	3.1–5.0	
Inner Mongolia	3.66%	3.1–5.0	
Shandong	3.98%	3.1–5.0	
Hainan	4.18%	3.1–5.0	
Sichuan	4.54%	3.1–5.0	
Yunnan	4.93%	3.1–5.0	
Ningxia	5.19%	5.1–35	Low-Level literate
Anhui	5.40%	5.1–35	
Guizhou	7.18%	5.1–35	
Gansu	9.11%	5.1–35	
Qinghai	9.16%	5.1–35	
Tibet	34.27%	5.1–35	

To measure the higher education efficiency, productivity, and regional heterogeneity in the technology of different groups for Chinese provinces, we use DEA Super-SBM, Meta-frontier analysis, and Malmquist productivity index, and results are presented in sections 4.1, 4.2, 4.3, and 4.4.

### 4.1 Super-SBM results

**Fig 1:**
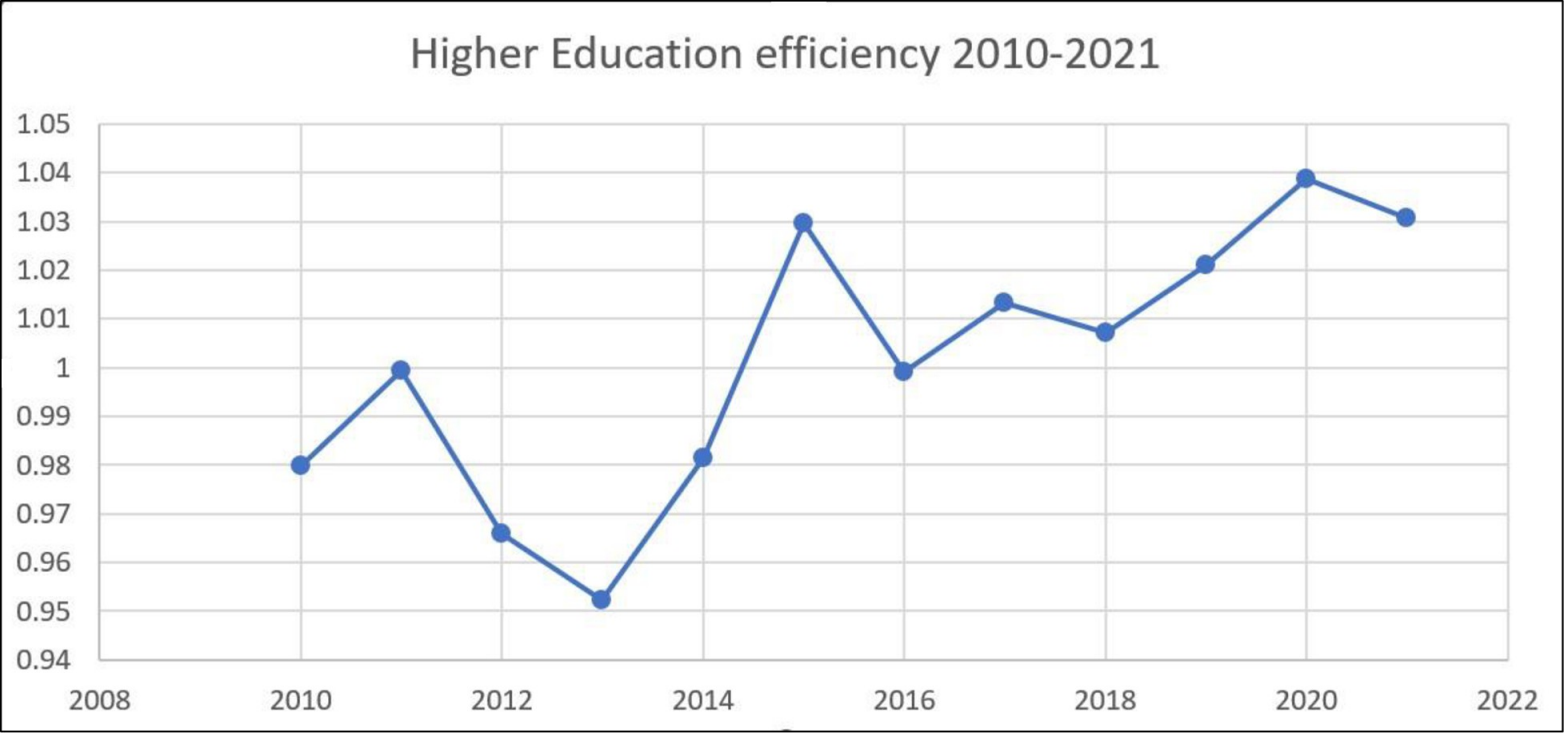
Higher education efficiency over the study period 2010–2021.

Fig 1 represents China’s higher education efficiency of China from 2010 to 2021. Higher education efficiency is a measure of the effectiveness with which resources are utilized in the higher education sector to generate desirable outcomes, such as successful graduation rates, research publications, and registrations of patents. It is frequently used to measure higher education institutions’ efficiency and productivity. Fig 1 presents the efficiency of the higher education sector of China. The values range from 0.9523 to 1.0387, with each value representing the year-specific efficiency level. A greater value indicates greater resource utilization efficiency in achieving intended outcomes. According to the data, there have been fluctuations in higher education efficiency. In 2020, for instance, efficiency peaked at 1.0387, indicating a highly productive year.

In contrast, 2013 has a lower efficiency of 0.9523, indicating that it was a relatively inefficient year for resource utilization. The average efficiency of higher education over the twelve years is 1.0015, indicating an upward trend in the efficiency of higher education over time. It is important to note that this average value does not necessarily imply that every year attained this exact efficiency level. Rather, it represents the average efficiency across all years in the table. Fig 1 illustrates a declining trend in higher education efficiency from 2010 to 2013, but after 2014, there was a rapid upward trend in higher education efficiency till December 2020. By analyzing this, one can gain insight into changes and trends in the efficiency of higher education over time, which can inform discussions and decisions regarding resource allocation, policy formulation, and enhancements to the higher education sector in China. The studies found that reducing the cost of inputs like inefficient funds distribution, reducing the human labor cost, and increasing the outputs through an efficient talent hunt for research output and quality graduates could increase the higher education efficiency in any particular system [81, 82]. Further researchers concluded that optimizing resource allocation in Brazilian higher education would require a 25% cost reduction, a 22% teacher reduction, and a 43% administrative staff reduction, given current attendance levels and GIC [83].

Moreover, student, regional, and managerial factors affect higher education efficiency. The study advises boosting student-teacher ratios and decreasing staff-teacher ratios to improve efficiency. The authors also suggest improving educational resource allocation and informing education sector public policy to boost higher education efficiency. Our Study results are aligned with the research output of [23, 24].

### 4.2 Meta frontier analysis results

Table 3 and S2 Fig detail the higher education resource technology gaps and the efficiency of higher education resources under Meta and group frontiers. Each year from 2010 to 2021 is included in the table, along with an average score. The "Meta frontier score" quantifies the optimal performance of Higher education regarding resource utilization. If the efficiency score exceeds 1, the allocated resources are used more effectively. A DMU’s "Group frontier score" indicates how well its resources perform compared to those of homogeneous DMUs (provinces). Similar to the Meta Frontier score, a number above 1 indicates higher education resource efficiency, while a score below 1 indicates resource inefficiency in the education system of a province. Differentiating between the Meta and Group frontier scores is represented by the "TGR" (Technology Gap Ratio). It measures how much better the top performers are than the group average. There is more room for improvement in resource efficiency if the TGR is low, which indicates a larger technological gap.

**Table 3:** Higher education resources efficiency under Meta, group frontier, and education resources technology gaps.

Year	Meta frontier Score	Group frontier Score	TGR
2010	0.9797	1.099	0.8891
2011	0.9993	1.0916	0.9141
2012	0.9658	1.0957	0.8786
2013	0.9523	1.0827	0.8781
2014	0.9814	1.0938	0.8944
2015	1.0295	1.0905	0.9443
2016	0.9991	1.0679	0.9325
2017	1.0132	1.0788	0.9371
2018	1.0071	1.0737	0.9371
2019	1.021	1.0741	0.9521
2020	1.0387	1.088	0.9555
2021	1.0307	1.0829	0.9522
Avg	1.0015	1.0849	0.9221

By analyzing the statistics, we can see how the use of resources in higher education has evolved. The Meta frontier scores indicate variations in education resource efficiency, which lie between 0.9523 and 1.0387. Scores between 1.0679 and 1.099 on the Group frontier indicate a level of efficiency that is typically above that of the group. There is a technology gap between the top-performing resources and the group average, as shown by the TGR values (which range from 0.8781 to 0.9555). The TGR readings, while not quite 1, nonetheless indicate a substantial technological difference. As evaluated by the Meta Frontier score, the average efficiency of resources used in higher education is 1.0015, greater than what would be considered optimal. With an average Group frontier score of 1.0849, the resources perform somewhat better than the average of the group as a whole. With a mean TGR of 0.9221, the performance difference between top and average resources is around average. In addition, the data in this table shed light on the technological diversity between the top and middle performers in higher education throughout the study period. The group frontier higher scores indicate that those provinces perform better in their group as compared to the comprehensive meta-analysis. Our study results are aligned with the finds of [84], who also recommend that production technology gaps in different regions of China should be minimized to optimize higher education resources.

Table 4 and S3 Fig compare provinces in terms of how efficiently they use their funding for higher education. Each province’s average score on the Meta-frontier, Group-frontier, and education resources technology gaps (TGR) is described in detail. Each province’s higher education "Meta-frontier score" reflects its potential to achieve maximum efficiency under optimal conditions. If the province’s score exceeds 1, its resources are being used more efficiently than other provinces. A lower score indicates less efficient use of those resources. A province’s "Group-frontier score" indicates how well this particular province performs in its group. If a province has an efficiency score higher than 1, its resources are more productive than average, whereas a lower number indicates resources are less productive than normal. Meta-frontier and Group-frontier scores are shown in the "TGR" (Technology Gap Ratio). For each state, it indicates the difference between the top performers and the group average. There is more room for improvement in resource efficiency if the TGR is low, which indicates a larger technological gap. Data analysis reveals regional differences in the efficiency of spending on higher education. Meta-frontier scores above 1 indicate resource utilization is higher than optimal in some provinces.

**Table 4:** Provincial higher education resources efficiency under Meta, group frontier, and education resources technology gaps.

DMU	Meta-frontier Score	Group-frontier Score	TGR
Anhui	1.0145	1.1634	0.8756
Beijing	1.2204	1.2208	0.9997
Chongqing	1.0314	1.102	0.9365
Fujian	0.7554	0.8492	0.8909
Gansu	0.9769	1.1862	0.8224
Guangdong	0.9392	0.9525	0.9831
Guangxi	0.9952	1.0483	0.9471
Guizhou	0.7366	1.0328	0.711
Hainan	1.1832	1.3012	0.9141
Hebei	0.8556	0.9089	0.9334
Heilongjiang	0.9393	1.0261	0.9145
Henan	1.1034	1.1904	0.9277
Hubei	1.1017	1.123	0.9809
Hunan	0.9563	0.9995	0.9541
Inner Mongolia	0.8957	1.0177	0.8761
Jiangsu	1.0397	1.0759	0.9669
Jiangxi	0.983	1.0861	0.9047
Jilin	0.9147	1.1075	0.8202
Liaoning	0.978	1.0173	0.9589
Ningxia	1.1192	1.1894	0.9416
Qinghai	1.1059	1.1227	0.9854
Shaanxi	1.1251	1.1996	0.9382
Shandong	0.9075	1.0354	0.8759
Shanghai	1.0802	1.0874	0.9935
Shanxi	1.0248	1.0903	0.9402
Sichuan	0.98	1.0332	0.9486
Tianjin	1.0671	1.1378	0.938
Tibet	1.1242	1.131	0.9941
Xinjiang	0.9763	1.0941	0.8913
Yunnan	0.8993	1.0358	0.8655
Zhejiang	1.017	1.0657	0.9543
**Avg**	**1.0015**	**1.0849**	**0.9221**

Qinghai (1.1059), Hainan (1.1824), and Beijing (1.2204) are only a few other examples. However, certain provinces, like Fujian (0.7554) and Guizhou (0.7366), have Meta-frontier values below 1, suggesting worse efficiency. Scores above 1 (such as Beijing’s 1.2208) indicate that a province’s resources are more efficient than the group average; scores below 1 (such as Guangdong’s 0.9525) indicate less efficiency than the group average. The Group-frontier scores also vary across provinces. The TGR numbers show how far each province is behind the group average regarding technology compared to the best-performing resources. If the value is less than 1, the technological gap is wider. Fujian (0.8910) and Guizhou (0.711) have lower TGR values than other provinces, indicating a high technological gap. As a whole, Chinese provinces have an average Meta-frontier score of 1.0015, which indicates that their higher education systems are slightly more efficient than they could be. The resources perform marginally better than the group average, as indicated by the Group-frontier score of 1.0849. The TGR value of 0.9221 indicates that there exists a moderate technology gap between the top-performing resources and the group average across provinces. In other words, the most efficient provinces in terms of technology utilization are moderately ahead of the average technological level within the entire group of provinces. Research studies have proved that technological gaps in any industry could impact the efficiency of that particular sector [38, 85].

Beijing, Hainan, and Shaanxi are the top three performers in the Meta frontier, while Hebei, Fujian, and Guizhou are the least efficient in higher education resource utilization. Similarly, Hainan, Beijing, and Shaanxi are the most efficient in the group frontier, and Guangdong, Hebei, and Fujian are the least efficient in resource utilization in their particular group frontier. Finally, the TGR of Beijing, Tibet, and Shanghai is closer to 1, indicating that these three provinces maintain higher technology utilized in the education sector of China. On the contrary, Gansu, Jilin, and Guizhou contain the least technology among all 31 provinces. Lytras et al. [86] discussed the importance of technology in higher education efficiency and its influencing factors on higher education efficiency. Therefore, our study advises the central government to set strategies for provincial governments to minimize the regional technological gaps in the country’s higher education sector to optimize the performance of DMUs.

**Fig 2:**
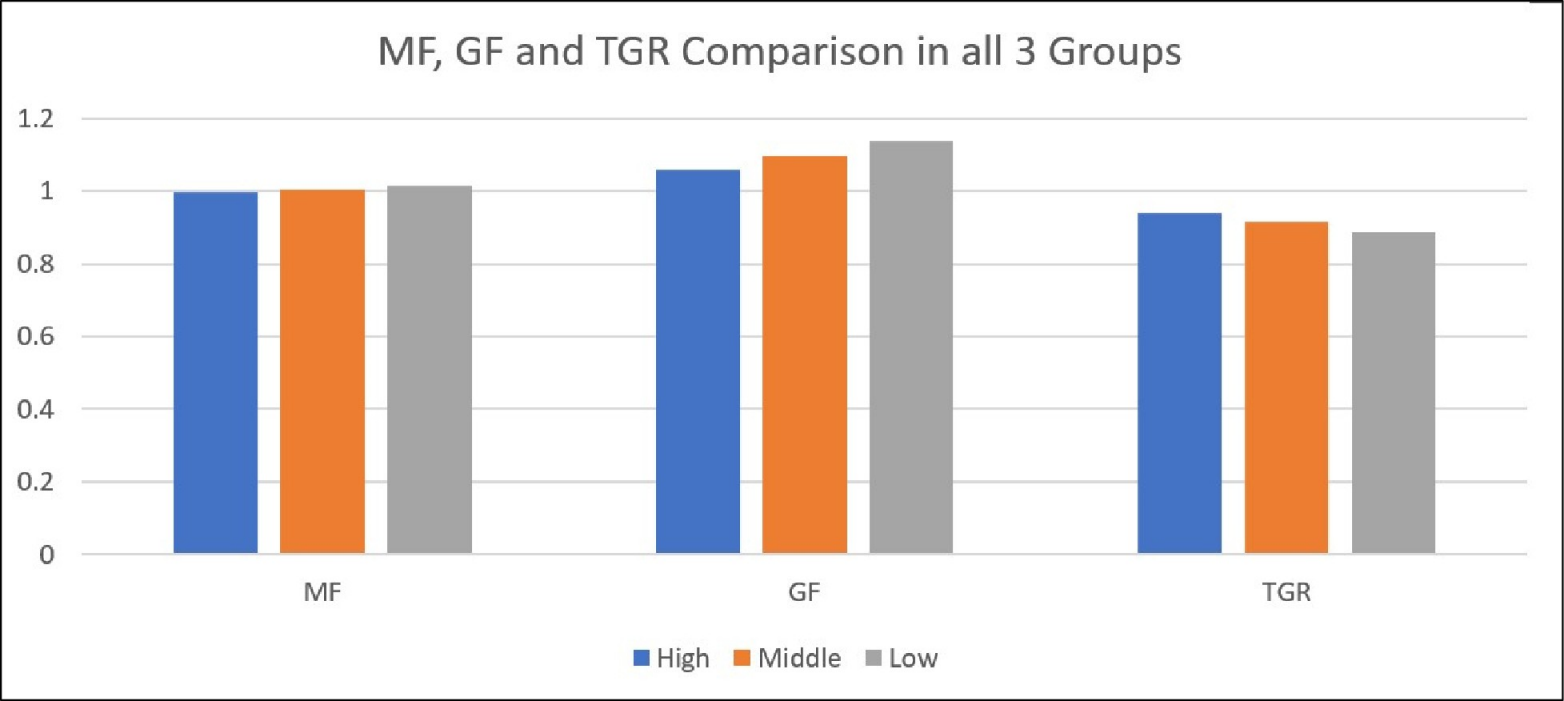
Comparison of high, middle, and low-level literate provinces for MF, GF, and TGR.

Fig 2 and S1 Table show the Meta frontier (MF), Group frontier (GF), and Technology Gap Ratio (TGR) scores for three groups classified according to their literacy levels. Those with a high level of education, a moderate level, and a low level of education. The average score for each category and the scores for particular regions within those categories are included. S1 Table displays the mean scores (MF, GF, and TGR) for the Highly-level literate group across multiple areas in China, including Beijing, Chongqing, Fujian, Guangdong, and others. These ratings show how well each region’s higher education resources perform compared to the ideal conditions depicted by the Meta frontier and how well they perform compared to the other regions in the same group, as shown by the Group frontier. TGR measures the performance gap between top resources and the group average across all regions. The group’s average scores are also revealed. The results can be used to compare the success of various locations and gauge the efficiency of their higher education systems. Results revealed that average efficiency scores of low-level literate provinces in group frontier and meta frontier are higher than those of middle and high-level literate provinces.

Further, middle-level literate provinces performed better than high-level literate provinces in meta and group frontier. These findings illustrate that Anhui, Gansu, Guizhou, Ningxia, Qinghai, and Tibet are more efficient in higher education resource utilization than their counterparts in middle and high-level literate groups. However, we found that the TGR score of high-level literate provinces on the technology gaps ratio is higher than middle and lower-level literate provinces. Comprehensive steps are needed to reduce China’s higher education technology gap ratio. It involves upgrading the internet, computer labs, and research facilities. Technology integration requires teacher training and assistance. In the digital age, bridging the gap and ensuring equal opportunities for students requires developing and distributing tailored digital content and resources, fostering collaboration and partnerships, establishing scholarships and financial aid programs, promoting research and development, and implementing supportive policies [87, 88].

### 4.3 Malmquist productivity index results

**Fig 3:**
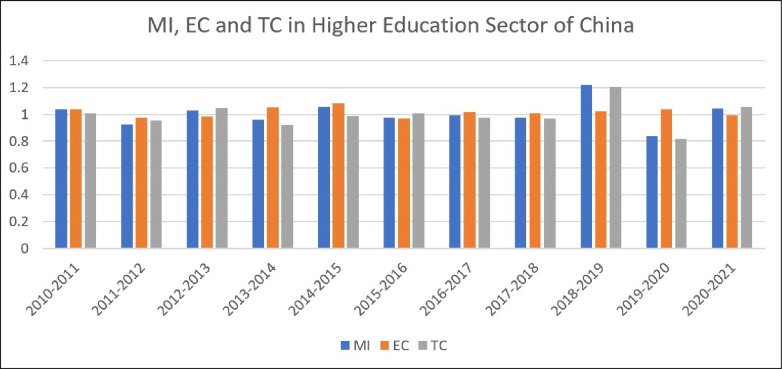
Average MI, EC, and TC scores in the higher education sector of China (2010–2021).

Fig 3 and S2 Table explain China’s higher education sector’s Malmquist Productivity Index (MI), Efficiency Change (EC), and Technology Change (TC) From 2010 to 2021. The table displays the values for MI, EC, and TC alongside columns for each year. On average, total factor productivity in the higher education sector has changed over the study period. The score of MI over one indicates the higher education Productivity incline, while decreases are illustrated by values below 1. For instance, the period of 2010–2011 shows that the MI score indicates a marginal improvement in productivity. However, the MI score in 2011–2012 (0.9233) demonstrates that productivity has fallen over the study period. The EC evaluates how the higher education sector of China as a whole has improved its resource utilization efficiency. If the value is greater than 1, then efficiency has increased, and if it is less than 1, efficiency has decreased. 2010–2011, for instance, the EC value was 1.0353, suggesting improved efficiency.

In contrast, the EC value in 2011–2012 was 0.9742, which indicates inefficiency. Similarly, TC reflects the development and evolution of technology used in the higher education sector. Increasing values over 1 indicate more advanced technology while decreasing numbers below 1 indicate less advanced technology. For example, the TC value for 2010–2011 is 1.0064, indicating a marginal technological capacity improvement. We get an average MI of 1.0034, an EC of 1.0157, and a TC of 0.9936. These results indicate that, on average, we have 0.34% growth in higher education productivity.

Further, EC is the main determinant of productivity growth as its value is higher than technology change (1.0157>0.9936). Explaining the results of EC and TC, we found a 1.57% growth in higher education e efficiency over the study period and a 0.63 percent decline in technological growth in the higher education sector of China. Fig 3 also explains the cause of higher education productivity changes each year. Higher education productivity is either associated with efficiency change or technology change. We found that dynamic change MI in most of the years is mainly due to a decline in technology change. Conversely, growth is associated with efficiency change for the study period. The results of the research study of Salleh [50] backed our findings and argued that National development and the knowledge economy depend on higher education’s productivity growth. AYRANCI [89] also applied the Malmquist Total Factor Productivity Index in the higher education sector in 21 OECD countries from 2000 to 2012. Results supported our finding that technology decline is the main cause of deterioration in higher education productivity. Australia, the USA, and Norway have the highest total factor productivity change indices, while New Zealand, the Czech Republic, and Turkey have the lowest. Total factor productivity rose the most in 2004–2005.

Table 5 and S4 Fig displays information about 31 Chinese provinces’ higher education sectors’ Malmquist Productivity Index (MI), Efficiency Change (EC), and Technology Change (TC). The table is broken down into three groups, labeled "highly literate," "middle-level literate," and "low-level literate," respectively, to reflect the varying levels of education in each location. Beijing, Chongqing, Fujian, Guangdong, Guangxi, Hebei, Heilongjiang, Henan, Hubei, Hunan, Jiangsu, Jilin, Liaoning, Shanghai, Shanxi, and Tianjin are only a few of the provinces represented in the Highly-level literate part of the table. The MI, EC, and TC values for each province are displayed. The Malmquist Productivity Index (MI) calculates the average annualized rate of change in total factor productivity. Table MI figures show the percentage of productivity increase or decrease from baseline. A score under 1 implies a decline in productivity, whereas a value above 1 shows growth.

**Table 5:** MI, EC, and TC in higher education sectors of 31 Chinese provinces.

Regions	DMU	MI	EC	TC
High-level literate	Beijing	0.9894	0.9991	0.9907
	Chongqing	1.0081	1.0049	1.0003
	Fujian	1.0086	0.998	1.0347
	Guangdong	1.1041	1.094	1.0564
	Guangxi	1.0523	1.0236	1.0319
	Hebei	0.9217	0.983	0.9399
	Heilongjiang	1.0031	1.0247	0.9815
	Henan	0.9853	0.9953	0.9902
	Hubei	0.9743	0.9934	0.9819
	Hunan	1.0028	1.0101	0.9998
	Jiangxi	0.9032	1.0058	0.8937
	Jilin	0.9855	1.042	0.9477
	Liaoning	0.9913	1.0113	0.9896
	Shanghai	0.9988	0.9988	1.0001
	Shanxi	1.023	1.0031	1.0201
	Tianjin	1.0039	0.9996	1.004
**Average**		**0.9972**	**1.01167**	**0.9914**
Middle-Level literate	Hainan	0.9761	0.9978	0.9742
	Inner Mongolia	0.9711	1.0777	0.9024
	Jiangsu	1.0274	1.0028	1.0246
	Shaanxi	1.0075	1.0083	0.9994
	Shandong	1.1106	1.0322	1.0749
	Sichuan	1.0425	1.0557	0.9992
	Xinjiang	0.9866	1.0371	0.9802
	Yunnan	0.9671	1.0383	0.9461
	Zhejiang	1.0225	0.9674	1.0644
**Average**		**1.0123**	**1.0241**	**0.9961**
Low-Level literate	Anhui	1.0017	0.9981	1.0037
	Gansu	0.9655	1.0085	0.9707
	Guizhou	1.1205	1.0752	1.0529
	Ningxia	0.954	0.9979	0.9554
	Qinghai	0.9987	1.0037	0.9955
	Tibet	0.9971	1.0004	0.9968
**Average**		**1.0062**	**1.0139**	**0.9958**
**Avg. 2010–2021**		**1.0034**	**1.0157**	**0.9936**

A rating of 0.9894 for Beijing indicates a modest decline in productivity, while a value of 1.1041 for Guangdong indicates growth. In the context of higher education, Efficiency Change (EC) measures how efficiently the province utilizes higher education resources. In the table, the EC values represent the efficiency shift in the first period. Efficiency increases for values greater than 1 and decreases for values less than 1. A rating of 0.9991 for Beijing indicates a slight decline in efficiency, while a value of 1.094 for Guangdong indicates growth. The ever-evolving nature of technology and the rapid pace of technical development are reflected in the term "Technology Change" (TC). The table’s TC values reflect the degree of technological advancement since the table’s initiation period. Technology improves when the value is greater than 1 and declines when it is less than 1. For instance, although Guangdong’s TC score of 1.0564 indicates technical advancement, Beijing’s 0.9907 indicates a modest technological decline.

The "Average" row in the Highly-level literate section provides average values for MI, EC, and TC across all provinces in this group. Inner Mongolia, Jiangsu, Shaanxi, Shandong, Sichuan, Xinjiang, Yunnan, and Zhejiang are all represented in the Middle-Level Literacy section. Data are presented similarly to the Highly literate part, matching MI, EC, and TC values. Anhui, Gansu, Guizhou, Ningxia, Qinghai, Tibet, and Hainan can all be found in the area devoted to people with low literacy levels. At the end of the table, in the "Average 2010–2021" row, the mean values for MI, EC, and TC across all provinces are shown during the period in question. The table summarizes productivity, efficiency change, and technological change in China’s higher education sector across provinces according to literacy rates. Table 5 further illustrates that higher education productivity change (MI) of middle-level literate provinces is higher than low-level and high-level literate provinces. A growth of 1.23 percent in the middle level and a decline of 0.28% was observed in high-level literate provinces. As EC is greater than TC in all three groups; therefore, we concluded that EC is the main determinant of growth in middle and low-level literate provinces, while TC decline is the cause of deterioration of productivity change in high-level provinces. Guizhou, Shandong, and Guangdong are the top three performers in higher education productivity growth, while Jiangxi, Hebei, and Ningxia are the least productive in higher education. Guangdong, Inner Mongolia, and Guizhou were more efficient over the study period as their EC was higher than the other remaining 28 provinces. Hubei, Hebei, and Zhejiang are the least efficient in using education resources. Finally, Shandong, Zhejiang, and Guangdong maintain superior technology with higher TC values; Hebei, Inner Mongolia, and Jiangxi are the lowest performers in the TC. Our findings are backed by some recent studies on productivity change in the higher education sector of China and concluded that technological change is the main cause of the productivity decline [90–92].

### 4.4 Kruskal Wallis test results

**Fig 4:**
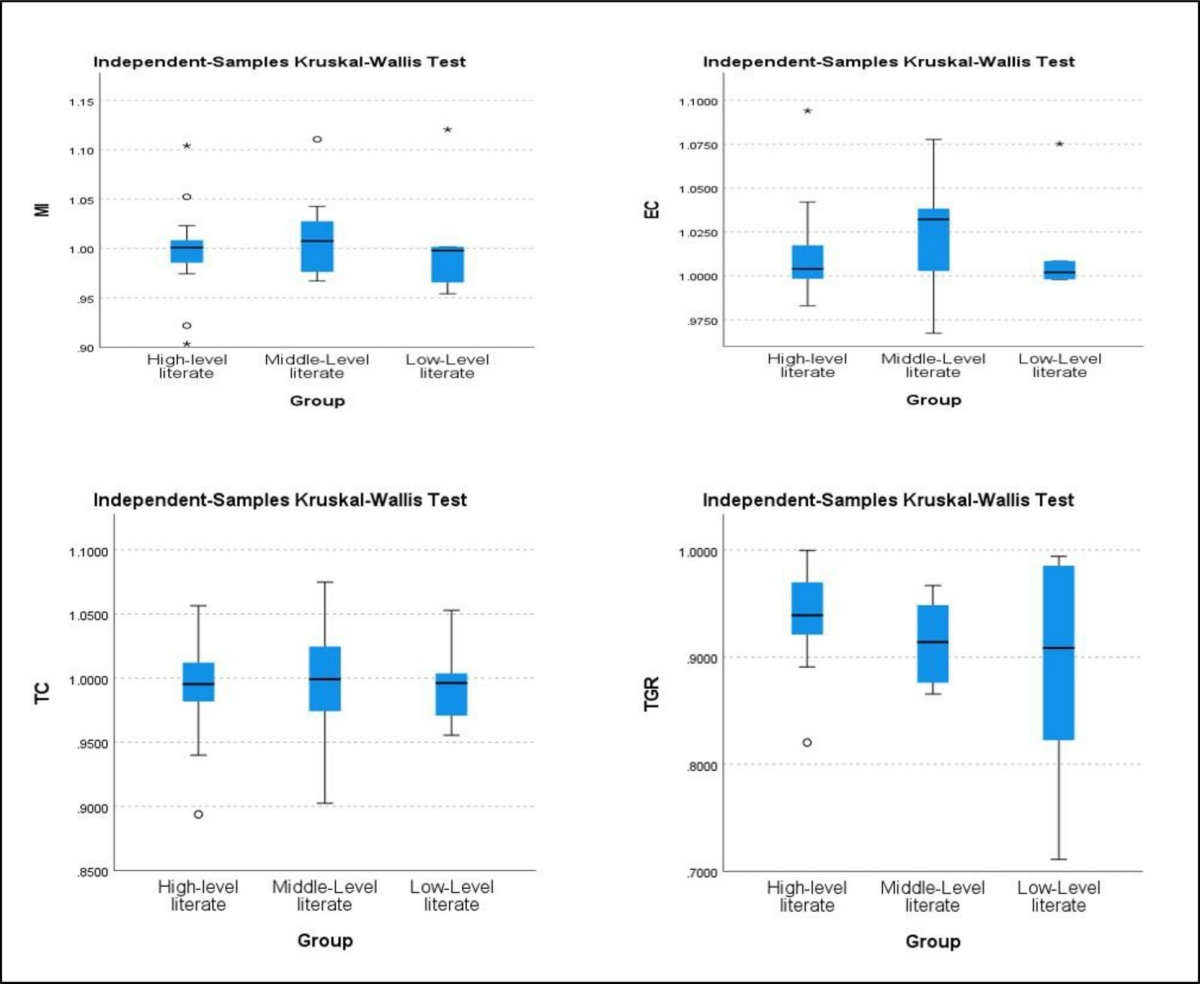
Kruskal-Wallis results for three different groups (education level) in China.

The findings from the three sections above illustrate that productivity change, efficiency change, technology change, and technology gap ratio within three distinct groups (high, middle, and low-level literate) exhibit variations based on the average values over the study period. To assess the statistical significance of these average scores, we conducted the Kruskal-Wallis test, which examines whether there are significant differences among the three education levels for MI, EC, TC, and TGR. Table 6 and Fig 4 present the results of the Kruskal-Wallis test, aiming to determine the potential statistical disparities in four variables (MI, EC, TC, and TGR) across the three Chinese education levels. Using a significance level of 0.050, each test’s null hypothesis assumes an identical score distribution across all education levels. The first test scrutinizes the MI score distribution across education levels, yielding a p-value of 0.002. This p-value falls below the significance level, leading to the rejection of the null hypothesis and indicating significant differences in MI scores across education levels. The second, third, and fourth tests on EC, TC, and TGR scores generate p-values of 0.000, 0.004, and 0.001, all of which are below the significance level. Consequently, these tests also reject the null hypothesis, signifying noteworthy score distinctions across education levels. All four Kruskal-Wallis tests reveal statistically significant variations across the three education levels in China concerning MI, EC, TC, and TGR scores. Therefore, to enhance higher education efficiency, productivity growth, and technological advancements, the central government should formulate policies aimed at reducing regional disparities and uniformly enhancing the quality of higher education institutions. This is in line with numerous research studies that support our findings, emphasizing the significant role regional disparities play in a country’s overall higher education inefficiency and declining productivity [93–95].

**Table 6:** Statistical differences for MI, EC, TC, and TGR in different groups of education levels.

Hypothesis Test Summary				
	**Null Hypothesis Test Sig. Decision**			
1	The distribution of MI is the same across categories of different groups.	Independent-Samples Kruskal–Wallis Test	.002	Reject the null hypothesis.
2	The distribution of EC is the same across categories of different groups.		.000	Reject the null hypothesis
3	The distribution of TC is the same across categories of different groups.		.004	Reject the null hypothesis
4	The distribution of TGR is the same across categories of different groups.		.001	Reject the null hypothesis

Asymptotic significances are displayed. The significance level is .050.

## 5. Conclusion and policy implications

The Chinese government invested heavily in the higher education sector of China to improve the quality of higher education institutions, minimize the higher education gaps in different regions, and improve the technology utilized in education in eastern coastal regions and the rest of the country. Moreover, numerous education policies were developed to efficiently utilize financial and human resources to increase universities’ and colleges’ quality of education and research output nationwide. Rapid economic growth also helped the government allocate sufficient budget resources to advance the education infrastructure in the country. To investigate the level of success in higher education efficiency and productivity growth of China, this research employed a set of methodologies to gauge the desired outcome. This research analyzes China’s provincial higher education systems’ efficiency, productivity, and regional technology gaps. DEA super-SBM, Meta-Frontier Analysis, and the Malmquist Productivity Index estimate higher education productivity and performance. China’s higher education system had an average efficiency (2010–2021) of 1.0015, demonstrating efficiency growth. From 2014 to 2020, higher education efficiency increased significantly, showing resource usage and management improvements.

In Meta-frontier and Group-frontier analyses, low-literate provinces had greater efficiency scores than intermediate and high-literate provinces. (TGR) show that high- and middle-literate provinces are technologically more advanced. Beijing, Hainan, and Shaanxi are the top three performers in the Meta frontier, while Hebei, Fujian, and Guizhou are the least efficient in higher education resource utilization. Similarly, Hainan, Beijing, and Shaanxi are the most efficient in the group frontier, and Guangdong, Hebei, and Fujian are the least efficient in resource utilization in their particular group frontier. Finally, the TGR of Beijing, Tibet, and Shanghai is closer to 1, indicating that these three provinces maintain higher technology utilized in the education sector of China.

On the contrary, Gansu, Jilin, and Guizhou contain the least technology among all 31 provinces. Malmquist Productivity Index scores average 1.0034, showing productivity growth over the research period. The data reveals efficiency change drives higher education productivity growth instead of technical progress. To boost productivity, higher education must improve operational efficiency and resource allocation. Finally, the Kruskal-Wallis test provides evidence that a significant statistical difference exists among the three groups of education levels for the average scores of MI, EC, TC, and TGR.

The study suggests numerous policy changes to improve China’s provincial higher education systems. Enhancing efficiency: To improve higher education efficiency, policy should promote efficient resource allocation, effective management, and institutional governance reforms. To close the technology gap between low-literate provinces, middle and high-literate provinces should share knowledge, collaborate, and transfer technology. Targeted support for low-literate provinces: Targeted investments, capacity-building programs, and partnerships with higher-performing provinces should address their specific challenges and improve their technological capabilities. Continuous assessment: To identify trends, evaluate policy actions, and inform higher education decision-making, efficiency, productivity change, and regional technological gaps should be monitored and evaluated regularly. Efficiency-driven reforms, such as process optimization, resource utilization improvement, and performance-based incentives for higher education institutions, should be prioritized because efficiency change drives productivity growth. These policy consequences can strengthen China’s provincial higher education institutions, promote regional equity, and boost productivity, boosting the sector’s development and competitiveness.

The study presents a comprehensive analysis of higher education efficiency and productivity in China’s provincial higher education systems. While it offers valuable insights, it has certain limitations, including potential data constraints, methodological assumptions, and limited generalizability. Future research in this field could extend the study’s findings by conducting more extended longitudinal analyses, integrating qualitative research methods, exploring international comparative studies, assessing the impact of policy changes, examining the role of technology in higher education, and conducting in-depth institutional case studies. These avenues for future research have the potential to provide a more nuanced and comprehensive understanding of the higher education sector, helping policymakers make informed decisions and further improving the efficiency and productivity of China’s provincial higher education institutions.

## Supporting information

S1 Fig Literacy level in China 2022. (DOCX)

S2 Fig Meta frontier, group frontier, and TGR (2010–2021). (DOCX)

S3 Fig Average meta frontier, group frontier and TGR in higher education of Chinese provinces (2010–2021). (DOCX)

S4 Fig MI, EC, and TC in higher education of Chinese provinces over the study period 2010–2021. (DOCX)

S1 Table Meta frontier, group frontier, and TGR scores for all 3 types of groups. (DOCX)

S2 Table MI, EC and TC in highereducation sector of China (2010–2021). (DOCX)
