# Supplementary figures and images for: Evaluating the efficiency, productivity change, and technology gaps of China’s provincial higher education systems: A comprehensive analytical framework
Source: PLoS One. 2024 Jan 19;19(1):e0294902. doi: 10.1371/journal.pone.0294902 (PMC10798458; doi:10.1371/journal.pone.0294902)

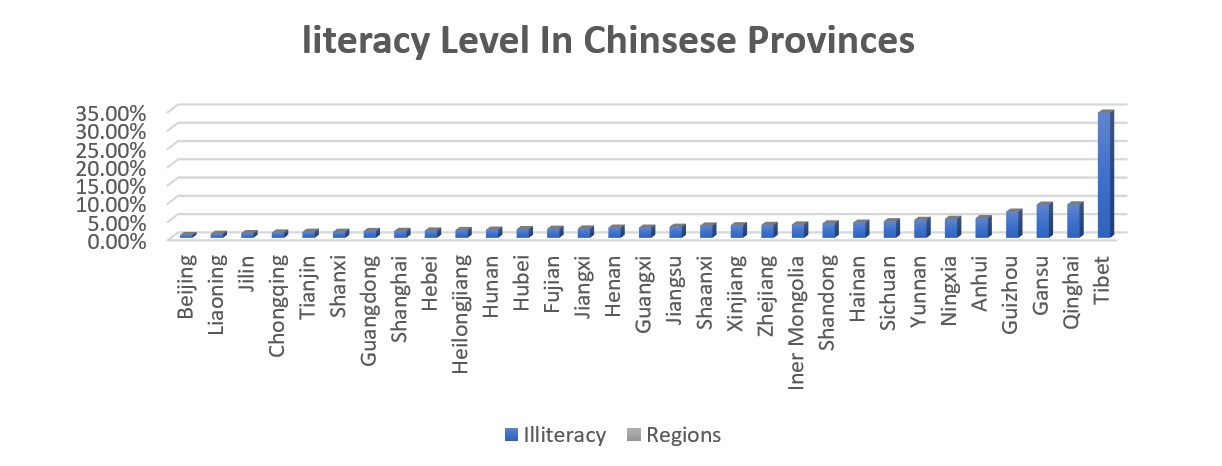

Supplement: S1 Fig — (DOCX) [file pone.0294902.s001.docx]

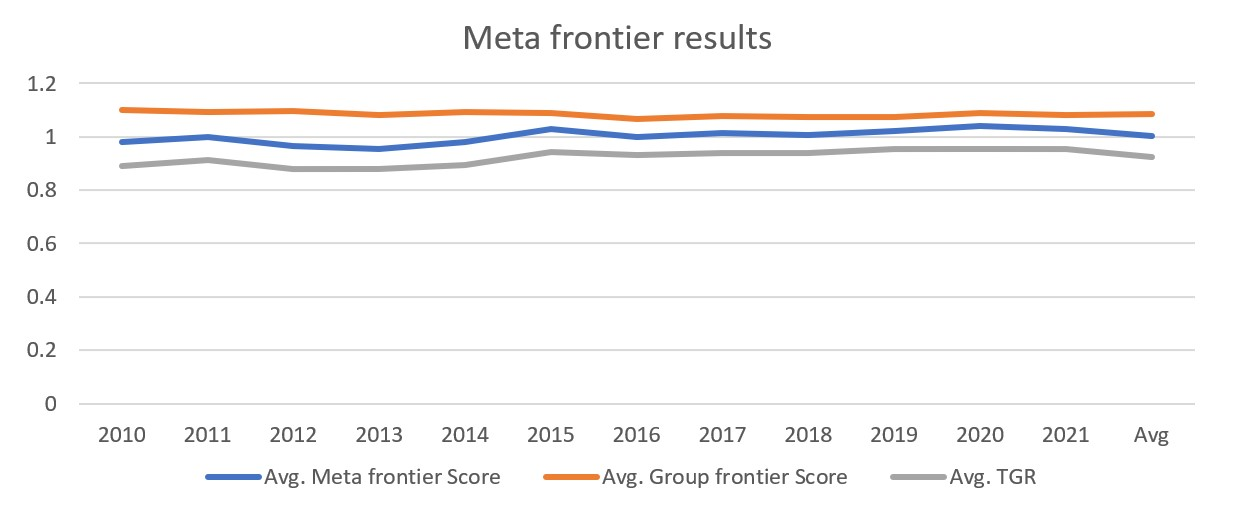

Supplement: S2 Fig — (DOCX) [file pone.0294902.s002.docx]

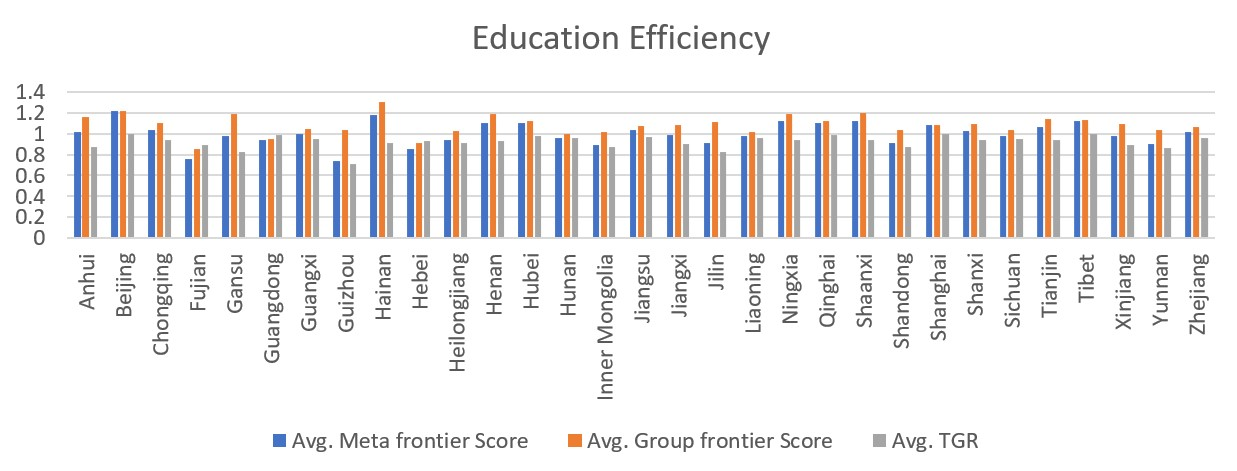

Supplement: S3 Fig — (DOCX) [file pone.0294902.s003.docx]

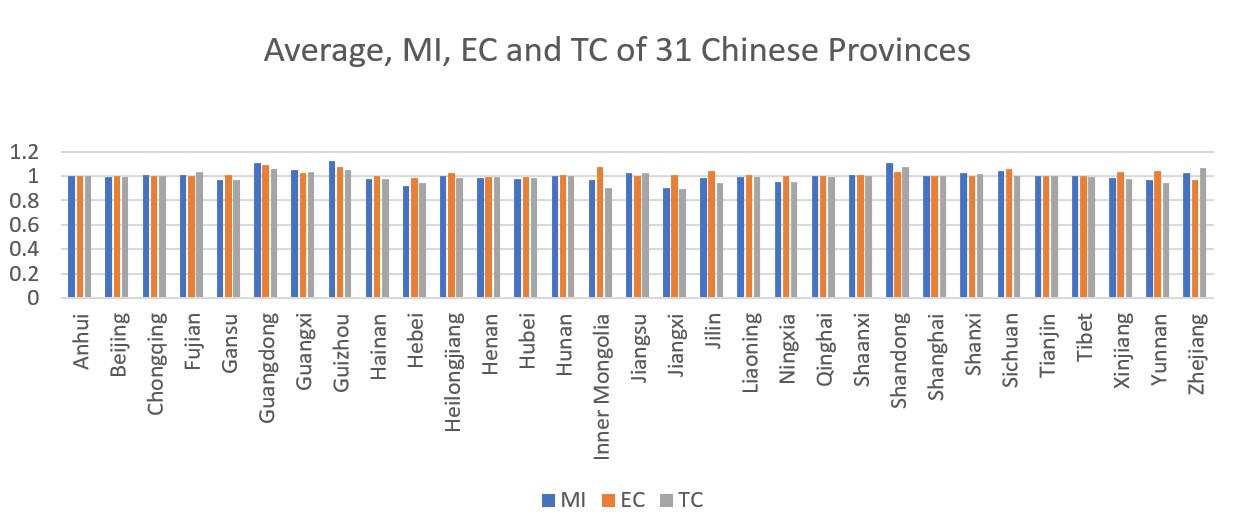

Supplement: S4 Fig — (DOCX) [file pone.0294902.s004.docx]
