# Supplementary material for: Evaluating the efficiency, productivity change, and technology gaps of China’s provincial higher education systems: A comprehensive analytical framework
Source: PLoS One. 2024 Jan 19;19(1):e0294902. doi: 10.1371/journal.pone.0294902 (PMC10798458; doi:10.1371/journal.pone.0294902)
Supplement: S1 Table — (DOCX) [file pone.0294902.s005.docx]

**Table A1.** Meta frontier, group frontier, and TGR scores for all 3 types of groups

| Regions | DMU | MF | GF | TGR |
| --- | --- | --- | --- | --- |
| High-level literate | Beijing | 1.2204 | 1.2208 | 0.9997 |
|  | Chongqing | 1.0314 | 1.102 | 0.9365 |
|  | Fujian | 0.7554 | 0.8492 | 0.8909 |
|  | Guangdong | 0.9392 | 0.9525 | 0.9831 |
|  |  |  |  |  |
|  | Guangxi | 0.9952 | 1.0483 | 0.9471 |
|  | Hebei | 0.8556 | 0.9089 | 0.9334 |
|  | Heilongjiang | 0.9393 | 1.0261 | 0.9145 |
|  | Henan | 1.1034 | 1.1904 | 0.9277 |
|  | Hubei | 1.1017 | 1.123 | 0.9809 |
|  | Hunan | 0.9563 | 0.9995 | 0.9541 |
|  | Jiangxi | 0.983 | 1.0861 | 0.9047 |
|  | Jilin | 0.9147 | 1.1075 | 0.8202 |
|  | Liaoning | 0.978 | 1.0173 | 0.9589 |
|  | Shanghai | 1.0802 | 1.0874 | 0.9935 |
|  | Shanxi | 1.0248 | 1.0903 | 0.9402 |
|  | Tianjin | 1.0671 | 1.1378 | 0.938 |
| **Average** |  | **0.9967** | **1.0591** | **0.9389** |
| Middle-Level literate | Hainan | 1.1832 | 1.3012 | 0.9141 |
|  | Inner Mongolia | 0.8957 | 1.0177 | 0.8761 |
|  | Jiangsu | 1.0397 | 1.0759 | 0.9669 |
|  | Shaanxi | 1.1251 | 1.1996 | 0.9382 |
|  | Shandong | 0.9075 | 1.0354 | 0.8759 |
|  | Sichuan | 0.98 | 1.0332 | 0.9486 |
|  | Xinjiang | 0.9763 | 1.0941 | 0.8913 |
|  | Yunnan | 0.8993 | 1.0358 | 0.8655 |
|  | Zhejiang | 1.017 | 1.0657 | 0.9543 |
| **Average** |  | **1.0026** | **1.0954** | **0.9145** |
| Low-Level literate | Anhui | 1.0145 | 1.1634 | 0.8756 |
|  | Gansu | 0.9769 | 1.1862 | 0.8224 |
|  | Guizhou | 0.7366 | 1.0328 | 0.711 |
|  | Ningxia | 1.1192 | 1.1894 | 0.9416 |
|  | Qinghai | 1.1059 | 1.1227 | 0.9854 |
|  | Tibet | 1.1242 | 1.131 | 0.9941 |
| **Average** |  | **1.0129** | **1.1375** | **0.8883** |
