# Supplementary material for: Evaluating the efficiency, productivity change, and technology gaps of China’s provincial higher education systems: A comprehensive analytical framework
Source: PLoS One. 2024 Jan 19;19(1):e0294902. doi: 10.1371/journal.pone.0294902 (PMC10798458; doi:10.1371/journal.pone.0294902)
Supplement: S2 Table — (DOCX) [file pone.0294902.s006.docx]

**Table A2.** MI, EC and TC in highereducation sector of China (2010-2021)

| Year | MI | EC | TC |
| --- | --- | --- | --- |
| 2010-2011 | 1.0374 | 1.0353 | 1.0064 |
| 2011-2012 | 0.9233 | 0.9742 | 0.9533 |
| 2012-2013 | 1.0252 | 0.9835 | 1.045 |
| 2013-2014 | 0.9579 | 1.0515 | 0.9187 |
| 2014-2015 | 1.0568 | 1.0823 | 0.9858 |
| 2015-2016 | 0.973 | 0.9694 | 1.0077 |
| 2016-2017 | 0.9905 | 1.0192 | 0.9709 |
| 2017-2018 | 0.973 | 1.0069 | 0.9691 |
| 2018-2019 | 1.2195 | 1.0236 | 1.2027 |
| 2019-2020 | 0.8373 | 1.0348 | 0.8149 |
| 2020-2021 | 1.0433 | 0.9925 | 1.0555 |
| Avg | 1.0034 | 1.0157 | 0.9936 |
